# Based on network pharmacology, molecular docking, and validation experiments to investigate the active components and mechanisms of action of Tibetan Medog County *Citrus medica L.*: In antioxidant activity

**DOI:** 10.1097/MD.0000000000045036

**Published:** 2025-10-17

**Authors:** Lin Tian, Pema Yang Zom, Chao Ma

**Affiliations:** a Tibet Plateau Institute of Biology, Lhasa, China.

**Keywords:** antioxidant, *Citrus medica L.*, molecular docking, network pharmacology

## Abstract

**Background:**

The antioxidant potential of citrus plants is closely related to their geographical origin, making it crucial to evaluate the natural antioxidant properties of *Citrus medica L. (C. medica*) from Medog County, Tibet.

**Methods:**

This study systematically investigates the antioxidant mechanisms of *C. medica* using network pharmacology, molecular docking, and experimental assays.

**Results:**

The antioxidant activity experiments showed that *C. medica* exhibits good bioactivity, and the fruit has better antioxidant activity than the leaves. Network pharmacology revealed 11 active components of *C. medica* with 1547 antioxidant-related targets. Key targets include TP53, IL6, AKT1, STAT3, and TNF. Gene ontology (GO) analysis identified 1419 biological process entries, 147 cellular component entries, and 306 molecular function entries. Kyoto Encyclopedia of Genes and Genomes analysis identified 212 antioxidant-related signaling pathways. The GO and Kyoto Encyclopedia of Genes and Genomes enrichment analyses showed that the targets are involved in cancer pathways, protein binding, enzyme binding, lipid metabolism, and atherosclerosis. Molecular docking demonstrated that the 11 active components of Medog *C. medica* exhibit binding energies with core targets TP53, IL6, AKT1, STAT3, and TNF generally less than −5 kcal·mol^−1^, indicating good affinity.

**Conclusion:**

This study identifies the excellent antioxidant activity of *C. medica* from multiple aspects and elucidates its potential antioxidant mechanisms, providing a theoretical basis for the development and application of *C. medica* as an antioxidant functional additive.

## 1. Introduction

Citrus medica L. (*C. medica*), a species belonging to the *Rutaceae* family, has been used in traditional Chinese medicine (TCM) for centuries, with applications in both medicinal and dietary contexts. Classical Chinese medicinal texts, such as Ben Cao Jing Ji Zhu, Xin Xiu Ben Cao, Ben Cao Shi Yi, Ben Cao Tu Jing, and the Chinese Pharmacopoeia, document its uses for treating abdominal distension, indigestion, vomiting, and cough.^[1,2]^ Beyond its traditional uses, modern pharmacological studies have revealed that *C. medica* demonstrates significant therapeutic effects in the treatment of bronchitis, hypertension, respiratory infections, cardiovascular diseases, and asthma.^[3]^

The fruit and leaves of *C. medica* are rich in plant secondary metabolites, which play a key role in biochemical and metabolic medicine and are composed of plant compounds such as phenolics, flavonoids, alkaloids, and terpenoids, which act through mechanisms such as regulating metabolic pathways, affecting gut microbiota, modulating immunity, anti-inflammatory effects, and antioxidant activities, and play an important role in the prevention and treatment of metabolic diseases such as obesity, diabetes, fatty liver, and cardiovascular diseases.^[4]^ The fruits and leaves of *C. medica* are rich in essential oils, which contain oxygenated derivatives, terpenoid hydrocarbons, alcohols, aldehydes, flavonoids, and other compounds.^[5]^ These bioactive components exhibit a wide range of biological activities, including anti-cancer, antioxidant, antibacterial, anti-inflammatory, blood sugar-lowering, and cardiovascular protective effects.^[6]^ Among these, flavonoids and phenolic acids are prominent antioxidants that enhance the body’s ability to counter oxidative stress.^[7]^ Network pharmacology, as an interdisciplinary field, integrates theories and methods from network science, systems biology, computer science, and other disciplines, aiming to comprehensively analyze the interactions between drugs and biological systems from a network perspective.^[8]^ Its core methodologies primarily include network construction, network analysis, and network simulation. By utilizing various biological entities, network pharmacology constructs interaction networks such as drug–target, drug–drug, and drug–disease networks, enabling the systematic elucidation of drug mechanisms of action and interactions among drugs. This approach not only enhances the efficiency and accuracy of drug research but also offers new insights and methodologies for drug development. Oxidative stress is a pathological state caused by the accumulation of reactive oxygen species (ROS), including free radicals and oxidative byproducts, which can arise from natural physiological processes, aging, excessive exercise, infections, or exposure to toxins. This imbalance can overwhelm the body’s antioxidant defenses.^[9,10]^ Consuming antioxidant-rich foods is crucial for neutralizing free radicals and preventing lipid peroxidation. Numerous studies have shown that citrus plants, including *C. medica*, exhibit significant antioxidant activity,^[11]^ and its fruit extract has demonstrated some degree of antioxidant potential.^[12,13]^ This study aims to experimentally evaluate the antioxidant activity of *C. medica* components and utilize network pharmacology to identify effective antioxidant substances and their molecular targets. By combining experimental assays and computational approaches, the study seeks to elucidate the antioxidant mechanisms of *C. medica* and explore its medicinal value and practical applications, with a focus on promoting its use in the food industry.

## 2. Materials and methods

### 2.1. Materials and instruments

The *C. medica* was collected from Motuo County, Nyingchi City, Tibet Autonomous Region, and the specimen is stored at the Tibet Plateau Institute of Biology. The following instruments and materials were used in the study:

Electronic analytical balance (JA2003); Steam distillation apparatus; New Century UV-Visible spectrophotometer; Micropipettes (Eppendorf); Ultrapure water system (ZYUPT-I-10T); Sodium chloride (analytical grade); DPPH (1,1-Diphenyl-2-picrylhydrazyl), Radical scavenging assay kit; 2,4,6-Tris(2-pyridyl)-s-triazine (TPTZ); Anhydrous ethanol (analytical grade); Methanol (chromatographic grade, Fisher); Ferric sulfate heptahydrate (analytical grade); Hydrochloric acid (analytical grade); Ammonium acetate; Ferric chloride; Salicylic acid; Hydrogen peroxide (analytical grade); and Anhydrous sodium sulfate.

### 2.2. Databases and software

The following databases and software were used in this study: Traditional Chinese Medicine Systems Pharmacology Database (TCMSP) (https://tcmsp-e.com/load_intro.php?id=39); SwissTargetPrediction platform (http://www.swisstargetprediction.ch/); Cytoscape 3.10.2 software (http://www.cytoscape.org/); PubChem Database (https://pubchem.ncbi.nlm.nih.gov/); DAVID Database (DAVID Functional Annotation Bioinformatics Microarray Analysis, https://davidbioinformatics.nih.gov/); OmicShare platform; STRING platform (version 11.0) (https://string-db.org/); Microbiology Information System (https://www.bioinformatics.com.cn); UniProt Database (https://www.uniprot.org/); STRING Database (https://cn.string-db.org); GeneCards Database (http://www.genecards.org/); PyMOL Visualization Software (https://pymol.org); AutoDock Analysis Software (https://autodock.scripps.edu); Venn Diagram Tool (http://bioinformatics.psb.ugent.be/webtools/Venn/); CTD Database (https://ctdbase.org).

## 3. Methods

### 3.1. Preparation of the test sample

The *C. medica* essential oil was extracted using the steam distillation method. The specific steps are as follows: Fresh *C. medica* peel and leaves (200 g each) were collected, crushed, and added to 1 L of distilled water for soaking for 5 hours. Then, the mixture was heated to boiling and distilled for 2 hours, with the volatile oil collected using ether. Anhydrous sodium sulfate was added to the extracted volatile oil, and the mixture was vigorously shaken until complete dehydration. The dehydrated essential oil was sealed and stored for later use.

### 3.2. GC-MS analysis

GC-MS conditions: Gas chromatography (GC) was performed using an HP-5MS fused silica capillary column (60 m × 0.32 mm × 0.25 μm); the injection port temperature was set at 250°C, with high-purity helium (>99.999%) as the carrier gas at a flow rate of 1.0 mL/min; samples were injected in splitless mode. The column temperature program was as follows: the initial temperature was set at 50°C and held for 2 minutes, then increased to 250°C at a rate of 5°C/min and held for 3 minutes, followed by an increase to 300°C at 10°C/min, with a total run time of 50 minutes. Mass spectrometry conditions: Electron ionization was used as the ion source at a temperature of 250°C with an ionization energy of 70 eV; the quadrupole temperature was set at 150°C, the interface temperature at 280°C, and the mass scan range was *m*/*z* 40 to 600. Compound identification was carried out by matching spectra with the NIST11 standard mass spectral library, and quantification was performed using the peak area normalization method to ensure the stability of the extract.

### 3.3. Measurement of antioxidant activity

#### 3.3.1. DPPH radical scavenging activity assay

This method was modified from a previously published protocol.^[14]^ Standard DPPH radical scavenging powder was dissolved in 80% methanol and subjected to gradient dilution. A standard curve was then constructed as follows:


$$\begin{array}{l} Standard\;DPPH\;radical\;scavenging\;rate\;\left(\%\right)=\left(1-\frac{A(S)}{A(S_{0})}\right)\times 100\% \end{array}$$

Where *S* represents the sample at various concentrations; *S*_0_ represents the control group with a zero concentration of the sample.

The *C. medica* sample was prepared into a stock solution, which was then diluted and quickly mixed with the DPPH solution. The absorbance was measured at 517 nm, and the DPPH radical scavenging rate of the sample was plotted.


$$\begin{array}{lll} & DPPH\;radical\;scavenging\;rate\;\left(\%\right)\; & =\left(1-\frac{\left(Absorbance\;of\;Sample\text{-}Control\right)}{Absorbance\;of\;Blank}\right)\times 100\% \end{array}$$

#### 3.3.2. *Hydroxyl radical scavenging activity assay*

This method was modified from a previously published protocol. First, a 8.8 mmol/L hydrogen peroxide (H_2_O_2_) solution was prepared and subjected to gradient dilution. Next, 1.0 mL of 9 mmol/L ferrous sulfate solution and 2.0 mL of 9 mmol/L salicylic acid ethanol solution were sequentially added to each gradient solution, and the mixture was thoroughly mixed. Then, the test sample (the sample with hydroxyl radical scavenging activity) was added to the reaction system. After mixing thoroughly, the sample was incubated in the dark at room temperature for 1 hour. After the reaction, the scavenging ability was measured at 510 nm using a microplate reader. Ascorbic acid (Vitamin C, Vc) was used as the positive control. The radical scavenging rate was then calculated.


$$\begin{array}{l} The\;hydroxyl\;radical\;scavenging\;rate\left(\%\right)=\frac{A_{0}-\left(A_{1}-A_{2}\right)}{A_{0}}\times 100\% \end{array}$$

Where *A*_0_ is the absorbance of the control; *A*_1_ is the absorbance of the sample with the test compound; and *A*_2_ is the absorbance of the sample without the test compound.

#### 3.3.3. *Ferric ion reducing antioxidant power (FRAP) assay*

Standard ferrous sulfate (FeSO_4_) solutions of concentrations 0.1, 0.2, 0.4, 0.6, 0.8, and 1.0 mmol/L were each mixed with 3 mL of FRAP working solution, and ultrapure water was added to achieve the desired final volume. The mixture was thoroughly mixed and reacted for 5 minutes. The absorbance was then measured at 593 nm using a spectrophotometer, with ultrapure water used as the blank. A standard curve was constructed. For the sample, 0.1 mL of the sample solution was added to 3 mL of FRAP working solution and 0.3 mL of ultrapure water. After thorough mixing, the reaction was allowed to proceed for 5 minutes. The absorbance was measured at 593 nm, with ultrapure water used to calibrate the spectrophotometer.

### 3.4. Mechanism of C. medica antioxidant effect based on network pharmacology

#### 3.4.1. Screening of C. medica active components

The chemical components of *C. medica* were identified using the TCMSP database with screening criteria of oral bioavailability (OB ≥ 30%) and drug-likeness (DL ≥ 0.18), supplemented by relevant literature. Structural diagrams of the compounds were retrieved from the PubChem database and imported into the SwissADME database for further screening. Active components were selected based on the following criteria: gastrointestinal (GI) absorption classified as “high,” and at least 2 “yes” results among the Lipinski, Ghose, Veber, Egan, and Muegge rules.^[15]^ Potential targets of these active components were predicted using the SwissTargetPrediction database (probability > 0) and the CTD database. The targets were refined and deduplicated via the UniProt platform, resulting in a final list of *C. medica* component targets.

#### 3.4.2. Antioxidant-related disease targets

Antioxidant-related gene symbols were collected by searching the keywords “Antioxidant” in the GeneCards, OMIM, and CTD databases. The results from these databases were merged, and duplicates were removed to yield a final list of antioxidant-related disease targets.

#### 3.4.3. Construction of the C. medica-antioxidant disease protein–protein interaction network

The intersection of *C. medica* component targets and antioxidant disease targets was determined to identify common targets. A Venn diagram was created to visualize the overlap. The common targets were input into the STRING database to obtain protein–protein interaction (PPI) data. The resulting network was constructed and analyzed using Cytoscape 3.10.2. Key targets were identified based on network parameters, including betweenness centrality, degree, and closeness centrality values, with thresholds set above their respective averages.

#### 3.4.4. GO functional and Kyoto Encyclopedia of Genes and Genomes (KEGG) pathway enrichment analysis

The common targets were analyzed using the DAVID database to perform gene ontology (GO) biological process enrichment analysis and KEGG pathway enrichment analysis.

#### 3.4.5. Molecular docking

The compounds identified through pharmacophore-based virtual screening were further evaluated by performing molecular docking into the active sites to analyze their binding interactions.^[16]^

The chemical structures of the active components were downloaded from the PubChem database. Core target proteins were identified based on their UniProt protein IDs, and their 3D structures were retrieved from the PDB database. Using PyMOL software, water molecules and native ligands were removed from the protein structures, which were subsequently prepared for docking by adding hydrogen atoms with AutoDock Tools. Active binding pockets were defined, and docking simulations between the proteins and small molecules were conducted using AutoDock Vina to compute binding energies. The docking results were visualized with PyMOL software.

## 4. Results

### 4.1. Comparison of antioxidant capacity of volatile oils from C. medica peel and leaves

#### 4.1.1. DPPH radical scavenging ability

**Figure 1.**
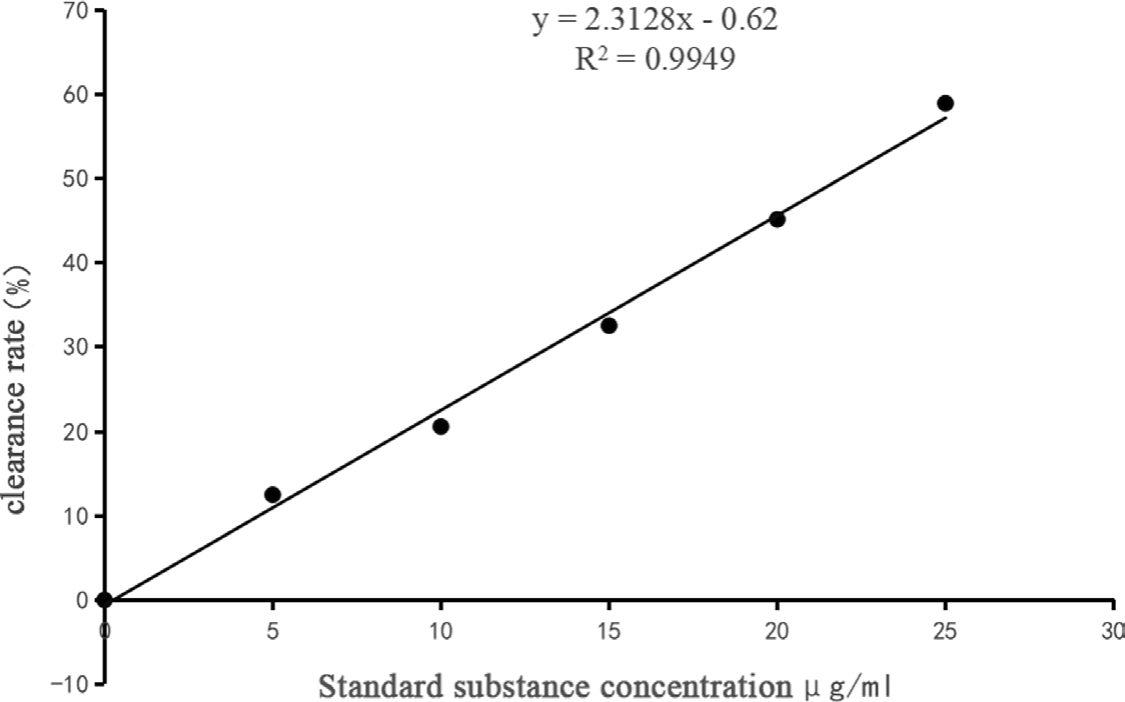
Standard curve of DPPH radical scavenging rate. DPPH = 1,1-diphenyl-2-picrylhydrazyl.


Table 1 presents the data used to construct the standard curve for DPPH radical scavenging rates. As shown in Figure 1, the standard curve exhibited a strong linear relationship. The regression equation derived from the data is:

**Table 1:** DPPH radical scavenging rates of standard solutions.

Standard solution concentration (µg/mL)	0	5	10	15	20	25
Absorbance	0.777	0.680	0.617	0.524	0.426	0.319
Scavenging rate (%)	0	12.48	20.59	32.56	45.17	58.94

DPPH = 1,1-diphenyl-2-picrylhydrazyl.

$$\begin{array}{l} Y=0.2231X+0.00062 \end{array}$$

where ***X*** represents the concentration of the standard solution (µg/mL) and ***Y*** represents the DPPH radical scavenging rate (%). The regression coefficient was *R*^2^ = 0.9949, indicating excellent linearity.

The scavenging rate (%) was calculated using the formula:


$$\begin{array}{l} Scavenging\;Rate\left(\%\right)=\left(1-\frac{\left(A\;\;\;Sample\text{-}AControl\right)}{A\;\;\;Blank}\right)\times 100\%, \end{array}$$

Table 2 presents The DPPH radical scavenging rates for the volatile components of *C. medica* peel and leaf essential oils were calculated to be 72.10% and 61.29%, respectively.

**Table 2:** DPPH radical scavenging capacity of volatile oils from *Citrus medica L.* peel and leaves.

Sample	Absorbance of control tube (nm)	Absorbance of test tube (nm)	Absorbance of blank tube (nm)	Scavenging rate (%)
Peel	0.441	0.653	0.760	72.10
Leaves	0.489	0.787	0.770	61.29

DPPH = 1,1-diphenyl-2-picrylhydrazyl.

#### 4.1.2. Hydroxyl radical scavenging ability

**Figure 2.**
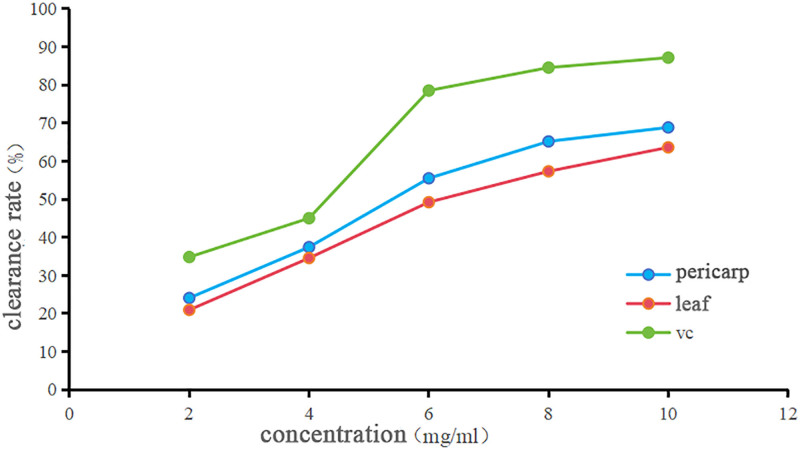
Comparison of OH scavenging ability. OH = hydroxyl radical.

Figure 2 presents the hydroxyl radical scavenging ability of *C. medica* volatile oils. The results indicate that the scavenging rates of the peel and leaf essential oils increased with concentration. Specifically, the scavenging rate of the peel oil rose from 24.06 to 68.83%, while the leaf oil increased from 20.92 to 63.60%. At concentrations of 2 to 6 mg/mL, the scavenging rates exhibited significant variation as the concentration increased. However, from 6 to 10 mg/mL, the rate of change became less pronounced. At the highest tested concentration (10 mg/mL), the scavenging rates for the peel and leaf volatile oils were 68.83% and 63.60%, respectively, highlighting their strong hydroxyl radical scavenging capacities.

#### 4.1.3. Ferric ion reducing antioxidant power

**Figure 3.**
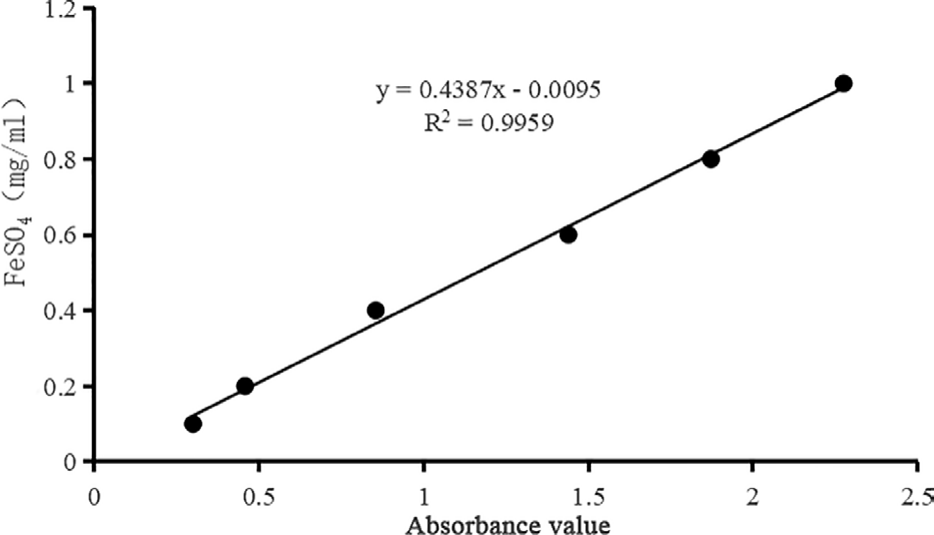
Standard curve for FRAP assay. FRAP = ferric ion reducing antioxidant power.


Figure 3 illustrates the standard curve for the FRAP assay, with the absorbance values of the FeSO_4_ solution plotted on the x-axis and the concentration of FeSO_4_ solution on the y-axis. The linear regression equation was determined as:

$$\begin{array}{l} Y=0.4387X-0.0095 \end{array}$$

where *X* represents the absorbance and *Y* represents the concentration of the FeSO_4_ solution (mg/mL). The regression coefficient (*R*^2^ = 0.9959) indicates a strong linear relationship and reliable calibration.

The antioxidant capacity of volatile oils from different parts of *C. medica* was measured using the FRAP method. The results, shown in Table 3, indicate that the reducing power of *C. medica* volatile oils from the peel was stronger than that from the leaves. The antioxidant results indicate that the fruit exhibits stronger antioxidant capacity than the leaves, which is consistent with the findings of Mateus et al.^[8]^

**Table 3:** FRAP values.

Sample No.	Extraction site	FRAP value (mean ± SD)
1	Peel	3.15 ± 0.72
2	Leaves	1.73 ± 0.05

FRAP = ferric ion reducing antioxidant power.

### 4.2. Prediction of the anti-inflammatory mechanism of C. medica in network pharmacology

#### 4.2.1. Prediction of disease-component common target genes and PPI network analysis

**Figure 4.**
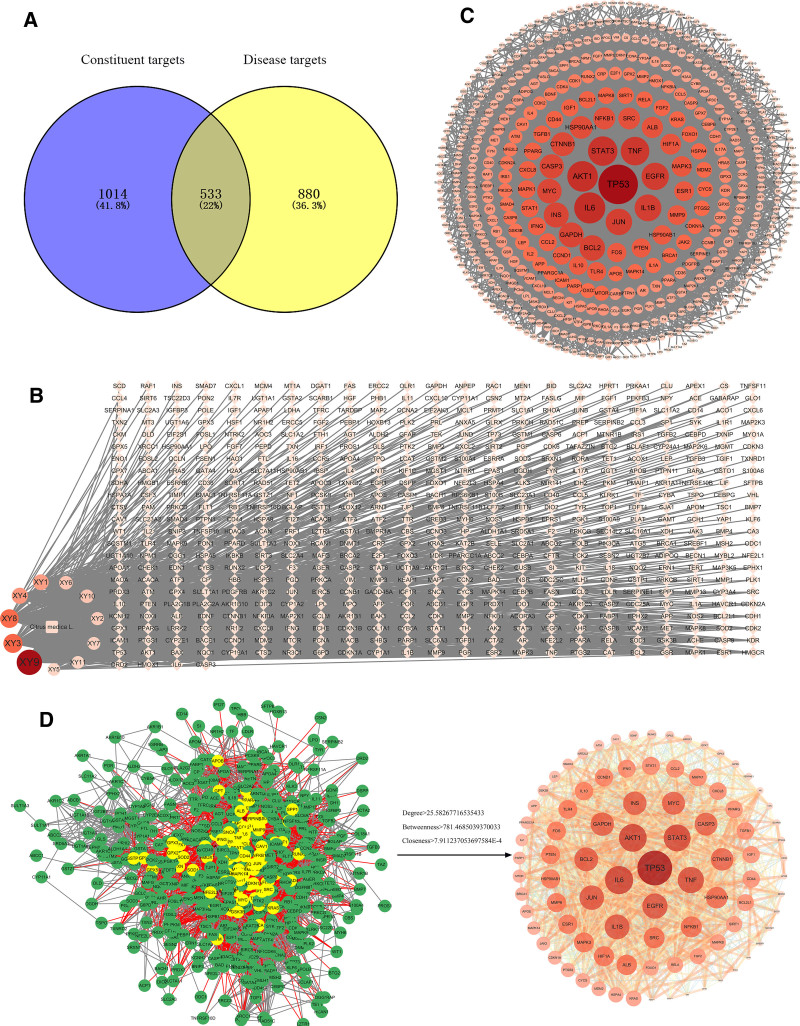
(A) Venn diagram of disease targets and compound targets. (B) Drug-component-disease-target network. (C) PPI network of intersection targets between *Citrus medica L.* and antioxidant targets. (D) core targets.

Based on the screening criteria described in section 2.4.1, a total of 11 compounds were identified (Table 4). Target prediction using the SwissTargetPrediction and CTD databases, followed by the removal of duplicates, resulted in 1547 compound-related targets. Concurrently, a search of the GeneCards and OMIM databases for targets associated with “oxidative stress” identified 1413 disease-related targets after deduplication. The intersection of these compound-related and disease-related targets yielded 533 common targets (Fig. 4A). These common targets were imported into the STRING database to analyze their interaction relationships and subsequently visualized using Cytoscape 3.10.2. The resulting “drug-active component-disease-target” network is shown in Figure 4B. Among the 11 compounds identified in *C. medica*, vitamin C, naringenin, linalool, hesperidin, and alpha-terpineol were most frequently associated with the common targets, suggesting they may serve as key active compounds in *C. medica*’s treatment of oxidative stress. The common targets were further analyzed in the STRING database, selecting “Homo sapiens” as the species and excluding isolated nodes. This produced a protein-protein interaction (PPI) network comprising 508 nodes and 6498 edges (Fig. 4C). Nodes with darker colors in the network indicate greater influence on the anti-inflammatory effect. Network feature analysis was performed, and targets with degree, betweenness, and closeness centralities exceeding their respective averages were identified as core targets. The thresholds were: Degree > 25.58267716535433, Betweenness > 781.4685039370033, and Closeness > 7.911237053697584E−4. This filtering resulted in 89 core targets, represented by 89 nodes and 1485 edges in the refined PPI network (Fig. 4D). The top 10 ranked targets, based on network centrality measures, are likely key mediators of *C. medica*’s anti-inflammatory effects. These genes include TP53, IL6, AKT1, STST3, TNF, EGFR, IL1B, JUN, BCL2, and GAPDH.

**Table 4:** Active compounds of *Citrus medica L.*

No.	Compound	MOLID	CAS
XY1	Linalool	MOL000198	78-70-6
XY2	Citral	MOL000124	5392-40-5
XY3	Naringenin	MOL004328	480-41-1
XY4	Hesperetin	MOL002341	520-33-2
XY5	Malic acid	MOL002299	6915-15-7
XY6	Limonin	MOL003959	1180-71-8
XY7	Terpineol	MOL000232	98-55-5
XY8	Geraniol	MOL000123	106-24-1
XY9	Vitamin C	MOL001691	50-81-7
XY10	Obacunone	MOL013352	751-03-1
XY11	Perilla aldehyde	MOL012895	2111-75-3

#### 4.2.2. GO functional and KEGG pathway enrichment analysis of related targets

**Figure 5.**
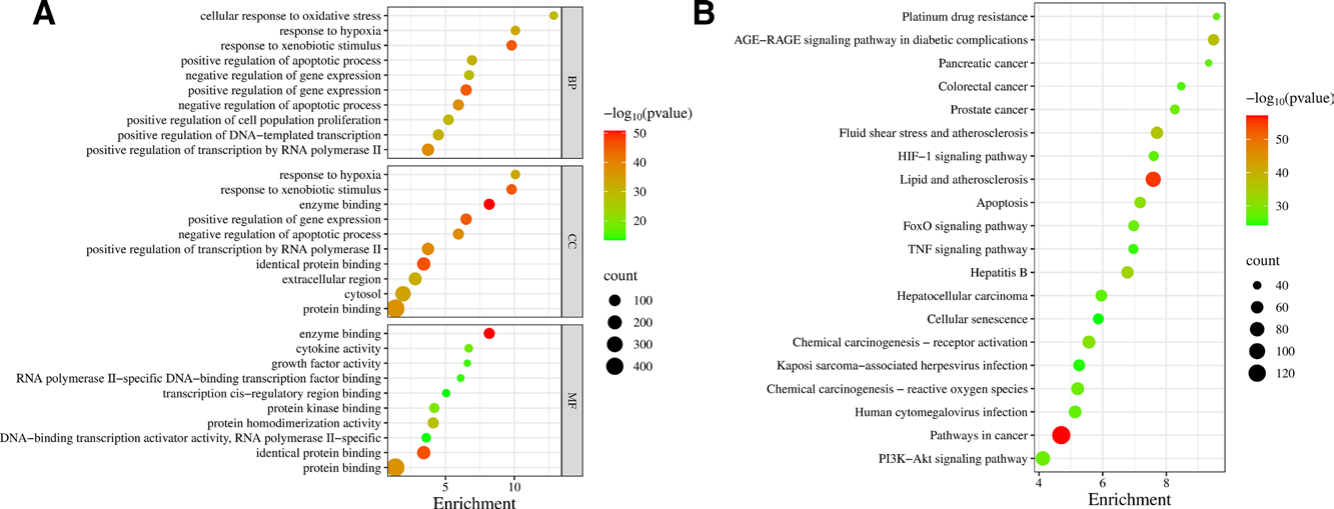
(A) GO enrichment analysis result chart. (B) Bar chart of GO annotation and KEGG analysis. GO = gene ontology, KEGG = Kyoto Encyclopedia of Genes and Genomes.

Functional annotation analysis of the 533 intersection targets related to the anti-inflammatory effects of *C. medica* was conducted using the DAVID database. The gene ontology (GO) analysis identified 1419 biological processes, 147 cellular components, and 306 molecular functions. The top 10 terms with the smallest p-values were selected for visualization (Fig. 5A). Key biological processes identified included protein binding, homophilic protein binding, enzyme binding, positive regulation of gene expression, and responses to external stimuli, indicating the diverse roles of these targets in biological systems. Similarly, KEGG pathway enrichment analysis of the 533 targets revealed 212 pathways. The top 20 pathways with the smallest p-values were selected for visualization (Fig. 5B). These pathways are closely linked to antioxidant and anti-inflammatory effects and include signaling pathways related to cancer, lipid metabolism, and atherosclerosis, as well as chemical carcinogen receptor activation, fluid shear stress, and the AGE-RAGE signaling pathway involved in diabetic complications. The enrichment results highlight that *C. medica* may exert its antioxidant effects through multiple mechanisms, including modulation of lipid metabolism, regulation of oxidative stress in chronic diseases, and influence on signaling pathways associated with inflammation and cellular stress responses.

#### 4.2.3. Molecular docking results

**Figure 6.**
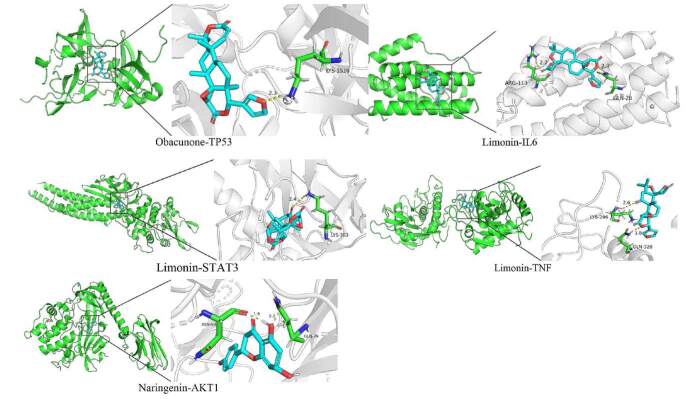
Molecular docking diagram of active components from *Citrus medica L.* with core targets.

The top 5 protein targets, identified based on their degree values in the network analysis, were selected for molecular docking with their corresponding potential active compounds. As shown in Table 5, a binding energy below −1.2 kcal/mol was considered indicative of relatively good binding activity. The larger the absolute value of the binding energy, the more stable the interaction between the active compound and the target receptor protein.^[17]^ The docking results demonstrated that the selected targets – TP53, IL6, AKT1, STAT3, and TNF – exhibited favorable binding affinities with their corresponding active compounds. Notably, Obacunone, Hesperetin, Naringenin, and Limonin were identified as having relatively strong binding affinities, making them potential key active compounds contributing to the antioxidant activity of *C. medica*. To further illustrate these findings, the docking interactions were visualized using PyMOL software, showcasing the spatial alignment and binding interactions of the compounds with the protein targets (Fig. 6). These results highlight the potential of these active compounds to stabilize key proteins involved in oxidative stress and inflammatory processes.

**Table 5:** Docking results of active components from *C. medica* with key targets.

No.	Protein target	Gene encoding	Active compound	Binding energy (kcal·mol^−1^)
1	TP53	6MXY	Linalool	−3.76
2		6MXY	Naringenin	−5.18
3		6MXY	Hesperetin	−5.93
4		6MXY	Geraniol	−4.77
5		6MXY	Vitamin C	−2.3
6		6MXY	Obacunone	−8.13
7	IL6	1ALU	Linalool	−3.42
8		1ALU	Citral	−3.37
9		1ALU	Naringenin	−4.62
10		1ALU	Hesperetin	−5.49
11		1ALU	Limonin	−6.67
12		1ALU	Geraniol	−4.1
13		1ALU	Vitamin C	−2.62
14		1ALU	Obacunone	−5.32
15	AKT1	8UW9	Linalool	−3.82
16		8UW9	Naringenin	−5.77
17		8UW9	Hesperetin	−3.32
18		8UW9	Limonin	−5.09
19		8UW9	Vitamin C	−2.65
20		8UW9	Obacunone	−5.88
21	STAT3	6NJS	Naringenin	−4.85
22		6NJS	Hesperetin	−3.91
23		6NJS	Limonin	−6.72
24		6NJS	Vitamin C	−2.99
25	TNF	3KME	Linalool	−5.27
26		3KME	Naringenin	−5.25
27		3KME	Hesperetin	−5.53
28		3KME	Limonin	−5.55
29		3KME	Geraniol	−3.68
30		3KME	Vitamin C	−2.31

## 5. Discussion

This study employed a combination of network pharmacology and experimental approaches to explore the antioxidant properties and mechanisms of *C. medica*. The findings demonstrate that *C. medica* exhibits strong antioxidant activity, driven by active compounds such as vitamin C, naringenin, hesperidin, linalool, and citronellol. This aligns with previous research highlighting the abundance of natural bioactive compounds in *C. medica*, which possess diverse biological activities, including anticancer, antioxidant, anti-hyperglycemic, antihypertensive, and anti-inflammatory effects.^[18,19]^ Despite these established benefits, the precise mechanisms underlying these activities remain incompletely understood. Somayeh Okhli et al demonstrated the stabilizing effects of *C. medica* essential oil on sunflower seed oil, outperforming synthetic antioxidants.^[20]^ Azhdarzadeh et al further reported that essential or volatile oils from citrus fruits are rich in phenolic compounds with significant antimicrobial and antioxidant properties.^[21]^ Pan Zhao et al used advanced analytical methods (TLC, HPLC-QTOF/MS, HPLC-DAD) to confirm the antioxidant activity of *C. medica*.^[22]^ Similarly, Mohadeseh Shojaemehr et al highlighted the antimicrobial and antioxidant properties of *C. medica* extracts.^[23]^

Key Active Compounds and Their Antioxidant Mechanisms Vitamin C, a potent antioxidant found in high concentrations in plants, serves as a redox buffer, reducing ROS, neutralizing oxidative free radicals, and mitigating oxidative stress.^[24,25]^ Its physiological roles include immune modulation, collagen synthesis, and neurotransmitter function; Naringenin, a flavonoid found in citrus fruits, has demonstrated strong free radical scavenging and anti-inflammatory activities. Rajappa Rashmi et al showed its potent free radical scavenging activity in vitro.^[26,27]^ while T. Annadurai et al confirmed its antioxidant and anti-hyperglycemic effects in diabetic models^[28]^; Linalool, a monoterpene with neuroprotective and antioxidant effects, has been shown to mitigate oxidative stress-induced neurotoxicity in experimental models^[29]^; Hesperidin, a traditional Chinese medicine compound, exhibits notable antioxidant and neuroprotective effects, enhancing antioxidant defenses via the ERK/Nrf2 signaling pathway and reducing oxidative damage.^[30,31]^

Molecular docking insights, molecular docking results revealed strong binding affinities between the key active compounds and critical antioxidant targets. For example, hesperidin demonstrated excellent binding affinity with TP53, IL6, AKT1, STAT3, and TNF, suggesting its potential role in modulating oxidative stress and inflammation. TP53, A tumor suppressor involved in ROS detoxification and apoptosis regulation.^[32]^ IL6, Combines autophagy with antioxidant responses, mitigating oxidative stress in β-cells.^[33,34]^ AKT1, Modulates cell survival and oxidative stress, influenced by SIRT1 overexpression.^[35,36]^ STAT3, Regulates redox homeostasis by modulating antioxidant enzymes.^[37]^ TNF, mediates oxidative stress-induced cell death.^[38,39]^

Mature fruits of *C. medica* are rich in medicinal and edible components, as well as a variety of essential trace elements required by the human body.^[40]^ Due to its excellent antioxidant activity, *C. medica* shows great potential for development, particularly in areas such as skincare product formulation. Its abundance of bioactive compounds also supports advances in pharmaceutical innovation and functional food development. Future research is recommended to focus on the in-depth exploration of its pharmacological mechanisms and clinical applications. However, the development and utilization of *C. medica* also face several challenges, such as low propagation efficiency, underutilization of byproducts, multi-step extraction processes with low yield of active constituents, and lagging development of high value-added products. The data in this study were collected from authoritative databases, the Chinese Pharmacopoeia, and relevant literature, ensuring mutual validation and reliable sources. This provides a solid foundation for further investigation into the antioxidant mechanisms and active substances of *C. medica* from Medog. It also offers a scientific reference for promoting the sustainable utilization of *C. medica* resources from Medog in the future.

## Author contributions

**Conceptualization:** Lin Tian.

**Data curation:** Lin Tian, Pema Yang Zom.

**Formal analysis:** Lin Tian.

**Funding acquisition:** Lin Tian.

**Project administration:** Chao Ma.

**Writing – original draft:** Lin Tian.

**Writing – review & editing:** Lin Tian.
